# Gonosomal Mosaicism for a Novel *COL5A1* Pathogenic Variant in Classic Ehlers-Danlos Syndrome

**DOI:** 10.3390/genes12121928

**Published:** 2021-11-29

**Authors:** Lucia Micale, Thomas Foiadelli, Federica Russo, Luigia Cinque, Francesco Bassanese, Matteo Granatiero, Carmela Fusco, Salvatore Savasta, Marco Castori

**Affiliations:** 1 Division of Medical Genetics, Fondazione IRCCS Casa Sollievo della Sofferenza, 71013 Foggia, Italy; f.russo@operapadrepio.it (F.R.); l.cinque@operapadrepio.it (L.C.); m.granatiero@operapadrepio.it (M.G.); c.fusco@operapadrepio.it (C.F.); m.castori@operapadrepio.it (M.C.); 2 Pediatric Clinic, IRCCS Policlinico San Matteo Foundation, University of Pavia, 27100 Pavia, Italy; t.Foiadelli@smatteo.pv.it (T.F.); francesco.bassanese01@universitadipavia.it (F.B.); salvatore.savasta@asst-crema.it (S.S.)

**Keywords:** classic Ehlers-Danlos syndrome, *COL5A1*, mosaicism

## Abstract

(1) Background: Classic Ehlers-Danlos syndrome (cEDS) is a heritable connective tissue disorder characterized by joint hypermobility and skin hyperextensibility with atrophic scarring. Many cEDS individuals carry variants in either the *COL5A1* or *COL5A2* genes. Mosaicism is relatively common in heritable connective tissue disorders but is rare in EDS. In cEDS, a single example of presumed gonosomal mosaicism for a *COL5A1* variant has been published to date. (2) Methods: An 8-year-old girl with cEDS was analyzed by next-generation sequencing (NGS). Segregation was performed by Sanger sequencing in her unaffected parents. In the father, the mosaicism of the variant was further analyzed by targeted NGS and droplet digital PCR (ddPCR) in the blood and by Sanger sequencing in other tissues. (3) Results: The NGS analysis revealed the novel germline heterozygous *COL5A1* c.1369G>T, p.(Glu457*) variant in the proband. Sanger chromatogram of the father’s blood specimen suggested the presence of a low-level mosaicism for the *COL5A1* variant, which was confirmed by NGS and estimated to be 4.8% by ddPCR. The mosaicism was also confirmed by Sanger sequencing in the father’s saliva, hair bulbs and nails. (4) Conclusions: We described the second case of cEDS caused by paternal gonosomal mosaicism in *COL5A1*. Parental mosaicism could be an issue in cEDS and, therefore, considered for appropriate genetic counseling.

## 1. Introduction

The Ehlers-Danlos syndromes (EDS) are a group of clinically varied and genetically heterogeneous hereditary soft connective tissue disorders mainly featuring doughy/velvety, soft, thin and/or variably hyperextensible skin; easy bruising with capillary fragility and joint hypermobility of various degrees. Additional features include skin fragility, abnormal wound healing and scarring formation, congenital contortionism, propensity to acquired dislocations and the fragility of vessels and internal organs [1]. The 2017 EDS classification describes 13 EDS types identified by clinical characteristics, inheritance pattern and molecular findings in 19 distinct genes [1]. Recent studies have described another genetically distinct classic-like EDS type caused by biallelic deleterious variants in the *AEPB1* gene [2], while additional cases have allowed to reclassify the so-called osteogenesis imperfecta/EDS overlap in *COL1*-related overlap disorder [1]. Classic EDS (cEDS; MIM #130000 and #130010) is presumably the most common Mendelian EDS type, with an estimated prevalence of ~1:20,000 in the general population [1]. Generalized joint hypermobility and skin hyperextensibility with atrophic scarring are the major hallmarks, whereas easy bruising, doughy skin, skin fragility, cutaneous pseudotumors, subcutaneous spheroids, abdominal wall hernias, epicanthal folds, complications of joint hypermobility and a positive family history are minor criteria [3].

Many cEDS individuals carry a heterozygous pathogenic or likely pathogenic variant in either the *COL5A1* or *COL5A2* genes, which encode for the type V collagen alpha 1 (α-1) and α-2 polypeptide chains, respectively [4,5,6]. Type V collagen occurs as heterotrimers of three different polypeptide chains: alpha (α)-1, α-2 and α-3, of which the different α-chains are encoded by *COL5A1, COL5A2* and *COL5A3* [7] or two copies of alpha-1 and one copy of alpha-2; it also occurs as a homotrimer of α-1 polypeptides. Each pro-α-chain comprises a triple-helical domain, consisting of repeating Gly-X-Y triplets flanked by globular amino (N-) and carboxy (C-) terminal propeptides. In a minority of cases, a classic-like EDS phenotype was molecularly resolved by the identification of the recurrent c.934C>T, p.(Arg312Cys) substitution in *COL1A1* or of biallelic causative variants in *TNXB*, *AEBP1* or *PLOD1* [6]. Nevertheless, about 18% of the cases of a cEDS phenotype remain without a proved molecular cause [6]. In these circumstances, the involvement of novel genes or atypical molecular mechanisms in known genes are evoked.

Mosaicism is often considered as an inheritance exception to the Mendelian laws, as well as a factor driving the intra- and interfamilial variability in autosomal-dominant traits. It is relatively common in collagen-related inherited disorders, such as osteogenesis imperfecta [8]. Nevertheless, genetic mosaicism has only rarely been documented in the 13 recognized types of EDS. In particular, a single example of presumed gonosomal mosaicism for a *COL5A1* pathogenic variant in cEDS has been published to date [9].

Here, we report a family with an affected girl and her unaffected father, the latter carrying the same *COL5A1* variant at the mosaic rate in the peripheral blood, hair bulbs, nails and saliva. We formally demonstrated the presence of the variant in the father’s tissues at a low rate, a finding confirming its mosaic origin. The transmission of the variant to the affected child documented gonosomal mosaicism.

## 2. Materials and Methods

### 2.1. Family Enrollment and Sample Preparation

The proband and her unaffected parents were enrolled in the routine activities of the Division of Medical Genetics at Fondazione IRCCS-Casa Sollievo della Sofferenza (San Giovanni Rotondo, Italy). Genomic DNA was extracted from proband’s and her parents’ peripheral blood leucocytes by using Bio Robot EZ1 (Quiagen, Solna, Sweden), according to the manufacturer’s instructions. For further investigation of the presumed mosaicism, genomic DNA was also isolated from proband’s and her father’s hair bulbs, nails and saliva by using commercial kit DNA IQ System (Promega Corporation, Madison, WI, USA), following different manufacturers’ protocols. The DNAs were quantified by a Nanodrop 2000 C spectrophotometer (Thermo Fisher Scientific, Waltham, MA, USA).

### 2.2. NGS Analysis 

First, the proband’s DNA underwent sequencing with a custom-made SureSelect gene panel (Agilent Technologies, Santa Clara, CA, USA) designed to selectively capture the known genes associated with a wide range of hereditary connective tissue and vascular/valvular disorders (“ConnectiveClinical” panel) and their major differential diagnoses, including: *ABCC6* (NM_001171.5), *ACTA2* (NM_001141945), *ADAMTS10* (NM_030957), *ADAMTS17* (NM_139057), *ADAMTS2* (NM_014244), *ADAMTSL2* (NM_014694), *ADAMTSL4* (NM_019032), *AEBP1* (NM_001129), *ALDH18A1* (NM_002860.3), *ATP6V0A2* (NM_012463.3), *ATP6V1A* (NM_001690.3), *ATP6V1E1* (NM_001696.3), *ATP7A* (NM_000052.6), *B3GALT6* (NM_080605), *B4GALT7* (NM_198540), *BGN* (NM_001711.5), *C1R* (NM_001354346), *C1S* (NM_001346850), *CBS* (NM_000071.2), *CECR1* (NM_001282225.1), *CHST14* (NM_130468), *COL11A1* (NM_001190709), *COL11A1* (NM_001854), *COL11A2* (NM_001163771), *COL12A1* (NM_004370), *COL1A1* (NM_000088), *COL1A2* (NM_000089), *COL2A1* (NM_001844), *COL3A1* (NM_000090), *COL4A1* (NM_001303110), *COL4A2* (NM_001846.3), *COL5A1* (NM_000093), *COL5A2* (NM_000393), *COL6A1* (NM_001848), *COL6A2* (NM_001849), *COL6A3* (NM_004369), *COL6A5* (NM_153264), *COL9A1* (NM_001851), *COL9A2* (NM_001852), *COL9A3* (NM_001853), *DLG4* (NM_001365.4), *DSE* (NM_001080976), *EFEMP2* (NM_016938), *ELN* (NM_000501), *EMILIN1* (NM_007046.3), *ENPP1* (NM_006208.3), *FBLN5* (NM_006329.3), *FBN1* (NM_000138), *FBN2* (NM_001999), *FKBP14* (NM_017946), *FLNA* (NM_001110556), *FOXE3* (NM_012186), *GATA5* (NM_080473), *GGCX* (NM_000821.6), *GLA* (NM_000169.2), *GORAB* (NM_152281.2), *HTRA1* (NM_002775.4), *LOX* (NM_002317), *LOXL3* (NM_032603), *LTBP2* (NM_000428), *LTBP3* (NM_001130144.2), *LTBP4* (NM_003573.2), *MAP3K7* (NM_145331), *MAT2A* (NM_005911), *MED12* (NM_005120.2), *MFAP5* (NM_003480), *MYH11* (NM_001040113), *MYH7* (NM_000257), *MYLK* (NM_053025), *NOTCH1* (NM_017617), *NOTCH2* (NM_024408.4), *NOTCH3* (NM_000435.2), *NT5E* (NM_002526.3), *PLOD1* (NM_001316320), *PRDM5* (NM_018699), *PRKG1* (NM_006258), *PTDSS1* (NM_014754.2), *PYCR1* (NM_006907.3), *RIN2* (NM_018993.3), *RYR1* (NM_000540), *SCN10A* (NM_006514), *SCN11A* (NM_014139),), *SCN9A* (NM_002977), *SEPN1* (NM_020451), *SGCB* (NM_000232), *SKI* (NM_003036), *SLC2A10* (NM_030777), *SLC39A13* (NM_001128225), *SMAD2* (NM_005901.5), *SMAD3* (NM_005902), *SMAD4* (NM_005359), *SMS* (NM_004595.5), *TAB2* (NM_015093), *TGFB1* (NM_000660.6), *TGFB2* (NM_001135599), *TGFB3* (NM_003239), *TGFBR1* (NM_001306210), *TGFBR2* (NM_001024847), *TNXB* (NM_019105), *TRIM37* (NM_015294), *TTR* (NM_000371), *UPF3B* (NM_080632.2), *YY1AP1* (NM_001198903.1), *ZDHHC9* (NM_016032.3) and *ZNF469* (NM_001127464). Libraries were prepared using the SureSelect target enrichment kit (Agilent Technologies, Santa Clara, CA, USA) following the manufacturer’s instructions. Targeted fragments were then sequenced on the NextSeq 500 platform (Illumina, San Diego, CA, USA) using a NextSeq 500 mid output kit V2.5 (300 cycles per flow cell). 

FastQC files were checked and trimmed, mapped reads recalibrated and processed and variants annotated by the Alissa Align & Call bioinformatics pipeline (Agilent Technologies, Santa Clara, CA, USA). Annotated variants were then filtered and interpreted with an internally implemented variant triage system by Alissa Interpret (Agilent Technologies, Santa Clara, CA, USA). Variants were first prioritized according to the following criteria: (i) nonsense/frameshift variant in genes previously described as disease-causing by haploinsufficiency or loss-of-function; (ii) missense variant with a REVEL score ≥ 0.70; (iii) variant affecting canonical splicing sites (i.e., ±1 or ±2 positions); (iv) variant absent in allele frequency population databases (dbSNP at https://www.ncbi.nlm.nih.gov/snp/ (accessed on 13 July 2021), gnomAD at https://www.gnomad-sg.org (accessed on 13 July 2021) and ExAC v0.3 at http://exac.hms.harvard.edu/, accessed on 13 July 2021/); (v) variant reported in allele frequency population databases, but with a minor allele frequency (MAF) lower than 0.05 and (vi) variant predicted and/or annotated as pathogenic/deleterious in ClinVar (https://www.ncbi.nlm.nih.gov/clinvar/ accessed on 13 July 2021) and/or LOVD (https://www.lovd.nl/, accessed on 13 July 2021) without evidence of conflicting interpretation. Selected variants were interpreted according to the American College of Medical Genetics and Genomics/Association for Molecular Pathology (ACMGG/AMP) [10]. 

Nucleotide variant nomenclature follows the format indicated in the Human Genome Variation Society (HGVS, http://www.hgvs.org, accessed on 27 September 2021) recommendations. The DNA variant numbering system refers to cDNA. Nucleotide numbering uses +1 as the A of the ATG translation initiation codon in the reference sequence, with the initiation codon as codon 1. Subsequently, a NGS analysis was also carried out on the father’s DNA in order to confirm and roughly quantify the percentage of the mutated allele, whose presence was originally supposed on Sanger sequencing (see below). 

### 2.3. Conservation of the Variant 

Evolutionary conservation of the p.Glu457 residue of the collagen alpha-1(V) chain was investigated with the protein sequence alignment generated by Clustal Omega (https://www.ebi.ac.uk/Tools/msa/clustalo/, accessed on 4 October 2021) and compared with the data provided by UC Santa Cruz Genome Browser 1.

### 2.4. Sanger Sequencing

Confirmation of the NGS data concerning the *COL5A1* candidate variant was first performed by Sanger sequencing on DNA extracted from the proband’s and her parents’ peripheral blood. Subsequently, Sanger sequencing was also carried out on DNA extracted from proband’s and her father’s hair bulbs, nails and saliva. For Sanger sequencing on DNA extracted from hair bulbs, nails and saliva, the experiment also included a control DNA extracted from the same tissues. The primers were designed by using primer3 tool (https://primer3.ut.ee/, accessed on 26 July 2021) to amplify *COL5A1* exon 9, checked by BLAST against the human genome to ensure its specificity (*COL5A1*_ex9_F: 5′-cctcctcgtctgaaggtgataa-3′, *COL5A1*_ex9_R: 5′-tgacttttccacaattctctagct-3′). The amplified products were subsequently purified by using ExoSAP-IT PCR Product Cleanup Reagent (Thermo Fisher Scientific, Wilmington, DE, USA) and sequenced by using a BigDye Terminator v1.1 sequencing kit (Thermo Fisher Scientific, Wilmington, DE, USA). The fragments obtained were purified using DyeEx plates (Qiagen, Tübingen, Germany) and resolved on an ABI Prism 3130 Genetic Analyzer (Thermo Fisher Scientific, Wilmington, DE, USA). Sequences were analyzed using Sequencer software (Gene Codes, Ann Arbor, MI, USA). The identified variant was resequenced in independent experiments. 

### 2.5. Digital Droplet PCR

DNA extracted from proband’s and her father’s peripheral blood, as well as from the peripheral blood of twenty healthy and unrelated controls, was used for further investigation aimed at (i) excluding false positive results in the father and (ii) more accurately quantifying the rate of the mutated allele in the latter. Samples were analyzed using the commercially Digital Droplet PCR (ddPCR) Mutation Detection Assay (ID: dHsaMDS322070417, Bio-Rad Laboratories, Hercules, CA, USA) to detect the *COL5A1* c.1369G>T variant and corresponding wild-type allele. Droplet generation, PCR cycling and droplet reading were performed according to the manufacturer’s recommendations. Briefly, DNA, labeled probes (hexachlorofluorescein (HEX) labels the wild-type allele, and fluorescein amidites (FAM) labels the mutant allele) and primers were mixed with 2x ddPCR Supermix for probes (Bio-Rad Laboratories, Hercules, CA, USA). Droplets were generated in the QX200 Droplet generator (Bio-Rad Laboratories, Hercules, CA, USA) and then transferred to a PCR plate and amplified in the PCR thermocycler. The conditions included enzyme activation at 95 °C for 10 min, followed by 40 cycles of denaturation at 94 °C for 30 s and annealing/extension at 55 °C for 1 min and enzyme deactivation at 98 °C for 10 min. After the reaction, the droplets were measured using the QX200 droplet reader. The measurements for method validation were performed in triplicate, and the data were reported as the mean. Fluorescence data were converted into concentrations according to the Poisson distribution statistical analysis using QuantaSoft Analysis software version 1.7.4.0917 (Bio-Rad Laboratories, Hercules, CA, USA).

## 3. Results

### 3.1. Case Report

An 8-year-old Caucasian girl was referred to our clinic for joint hyperlaxity, skin hyperextensibility and delayed wound healing. She was the second child of non-consanguineous parents, born preterm (29 weeks + 6 days) with an urgent Cesarean section due to maternal pre-eclampsia and placental abruption. Birth weight was low but appropriate for gestational age (930 g; 11th centile), and prematurity requested prompt admission to the neonatal intensive care unit. Twelve hours after birth, she experienced small bowel perforation due to meconium ileus, which required resection surgery and subsequent ileostomy without local complications. In the subsequent weeks, bilateral retinal detachment likely due to the retinopathy of prematurity was also diagnosed and promptly treated with laser photocoagulation and subsequent vitrectomy at 2 months of age. Additionally, she was diagnosed with bilateral cataract presumably secondary to prematurity. For this complication, she underwent surgery by the age of 18 months and 3 years to the left and right eye, respectively. The ophthalmologic prognosis was complicated by high-grade myopia and visual deficit. According to the last evaluation, she had a visual acuity of 3/10 in the left eye and a partial blindness in the right one (she only perceives lights), treated with daily topic ocular β-blockers. At the age of 7, she had a right traumatic femoral bone fracture after a minor trauma (a fall from a chair), requiring surgical treatment.

**Figure 1:**
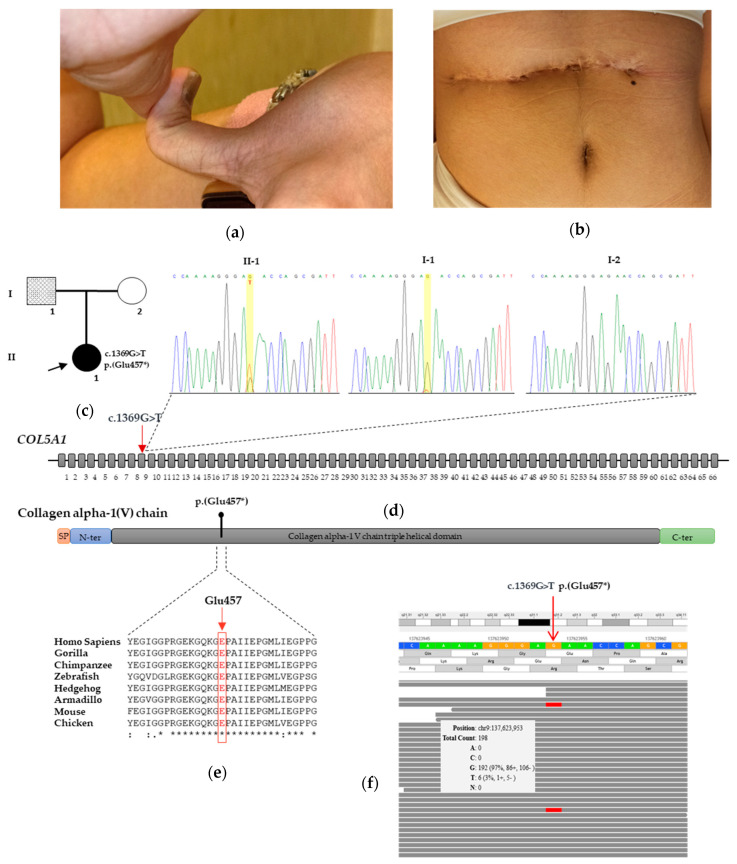
Clinical aspects and molecular findings. (**a**) Distal phalanx hyperextension of the thumb in the proband. (**b**) Dystrophic post-surgical scarring formation in the proband. (**c**) Pedigree of the studied family with parental mosaicism; the affected heterozygous proband is indicated with a filled symbol, and the individual with mosaicism is indicated by a hatched symbol. (**d**) Electropherogram showing DNA sequencing of the proband carrying c.1369G>T, p.(Glu457*) in exon 9 of *COL5A1* and her unaffected parents. In the middle of the panel, there is a schematic representation of the *COL5A1* gene that shows the heterozygous c.1369G>T variant. A yellow bar indicates the affected nucleotide. The arrow indicates the germline variant in the proband. The identified variant is also put in a gene and protein context. In the middle, the coding regions are in grey; the black line represents the intron regions that are not to scale. In the lower part is a schematic diagram of the collagen type V alpha-1 chain protein: the signal peptide is in red, and N-terminal and C-terminal propeptide domains are in blue and green, respectively. (**e**) Alignment of the amino acid sequence of the collagen type V alpha-1 chain region, including the residue p. Glu457 among several species, was generated by Clustal Omega. (**f**) Genomebrowser view from the Alissa software output. The grey window shows that the *COL5A1* c.1369G>T allele is present in 3% of the reads in the father’s blood specimen.

On examination, the girl was found to be overweight (weight 75–90th centile; BMI 75th centile—CDC charts [11], with generalized joint hypermobility (Beighton score: 9/9) (Figure 1a), skin hyperextensibility, multiple atrophic and post-surgical dystrophic scars (Figure 1b), multiple ecchymoses in her lower limbs, absence of lingual frenulum, mild right-convex thoracic scoliosis, bilateral genu valgum-recurvatum, cubitus valgus with elbows hyperextensibility and bilateral pes planus. She had no strength deficit, with global hypotonia but normal muscular trophism and deep tendon reflexes. However, she had a global hypotonia. Intellectual abilities were normal. She was able to walk, with the help of a crutch. On both legs, soft, velvety skin and subcutaneous tissues had been long misdiagnosed as a mild lymphedema. Parents also reported easy bruising for their daughter. None of her family members presented with similar signs or symptoms. Chest, spine and limb radiography confirmed the orthopedic abnormalities. Global respiratory function with spirometry, abdominal and supra-aortic trunk ultrasounds and video-electroencephalography all resulted normal. A comprehensive cardiovascular evaluation evidenced a mild mitral valve insufficiency without clinical relevance. No other vascular or lymphatic anomalies were detected. Multidisciplinary follow up, including pediatric, oculistic, psychiatric and cardiovascular evaluations, has been continued for 5 years. She reports no pain. She attends school with good cognitive and social skills and weekly swimming sessions. Support insoles were prescribed without a clear clinical improvement. 

### 3.2. Molecular Findings

In the proband, the molecular analysis revealed the heterozygous nonsense c.1369G>T variant located in exon 9 of *COL5A1*. This variant was predicted to incorporate a premature stop codon (PTC) at the evolutionarily conserved residue 457 (p.(Glu457*)) (PVS1_Very Strong criterion) [10] (Figure 1c,d,e). It was absent from all the applied population and disease-specific databases, including dbSNP, ExAC, 1000 Genomes and gnomAD, suggesting that it is novel (PM2_Moderate criterion) [10]. The observed phenotype (see “case report”) was highly specific for *COL5A1* (PP4_Supporting criterion). Therefore, *COL5A1* c.1369G>T, p.(Glu457*) was interpreted as “pathogenic”. Blood samples from the patient’s parents were also screened by direct Sanger sequencing. Although there was no evidence of the variant in the mother, a very small peak was observed in the Sanger chromatogram of the father’s blood specimen, which suggested the presence of a genetic mosaicism (Figure 1c). This data was also detected during independent DNA extraction. Careful clinical examination did not find any skeletal, skin or other diagnostic criteria in the father. 

**Figure 2:**
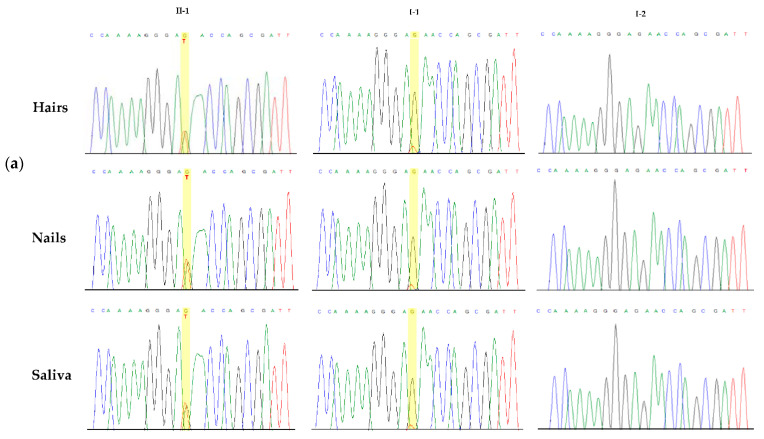

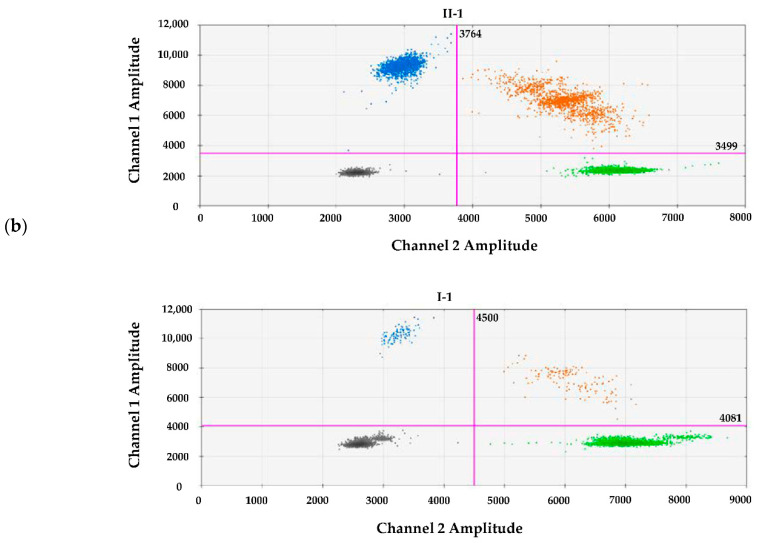
*COL5A1* c.1369G>T mosaicism among different tissues, and quantification of the mutant frequency alleles in the blood samples. (**a**) Electropherograms showing Sanger sequencing of *COL5A1* exon 9 harboring the c.1369G>T, p.(Glu457*) variant in the hairs, nail bulbs and saliva of the proband, her father and a healthy individual. (**b**) The ddPCR analysis output shows two-dimensional scatter plots in which Channel 1 fluorescence (FAM) is plotted against Channel 2 fluorescence (HEX) for each droplet. The droplet populations are indicated as follows: double-negative (grey), FAM-positive (blue), HEX-positive (green) and double-positive (brown = positive for FAM and HEX in the same droplet).

To confirm the mosaic state of the variant in the father’s DNA, we employed a targeted NGS analysis, including the *COL5A1* gene, which offers a better sensitivity to detect mosaic variants than direct sequencing. The NGS analysis demonstrated that the mutant allele was present in no less than 3% of the reads in the father’s blood (Figure 1f). The variant was also confirmed by Sanger sequencing at a low but visible rate in other tissues of the patient’s father, including saliva, hair bulbs and nails (Figure 2a). This collective evidence suggested that the mosaic variant in the father arose as a post-zygotic mitotic event and was an example of gonosomal mosaicism. 

Given the known different sensitivities for mosaicism of Sanger sequencing and NGS, we further verified the *COL5A1* variant and its rate in the father’s tissues by a ddPCR analysis. The ddPCR analysis revealed that the frequency of the mutant allele was 4.8% in the father’s peripheral blood, while it was 50% in the proband, as expected for a germline variant. Consistently, the controls had zero mutant frequency, signifying a wild type on both alleles (Figure 2b). The above results confirmed the low-level mosaicism of the *COL5A1* c.1369G>T variant in the father and its borderline nature in relation to the Sanger sequencing sensibility. The novel variant has been submitted to LOVD (ID: 0000814510). 

## 4. Discussion

*COL5A1* variants are scattered throughout the gene, and the majority of them lead to COL5A1 haploinsufficiency, usually resulting from nonsense-mediated mRNA decay (NMD) [4,5,6]. Variants located at the signal peptide or C-propeptide of proα1(V) reduce type V procollagen secretion, and this corroborates the hypothesis that “functional” type V collagen haploinsufficiency is a crucial factor in cEDS pathogenesis. For the remaining, less frequent, pathogenic variants—typically, in-frame exon skips and helical glycine substitutions—the exact mechanism of action is yet unknown, but they might interfere with the secretion and incorporation of type V collagen molecules into fibrils [12]. 

Here, we identified the novel *COL5A1* c.1369G>T variant in an 8-year-old Caucasian girl with full-blown cEDS. The nucleotidic change fell in exon 9 and was predicted to introduce a PTC in the triple-helical domain at position 457. As the PTC maps at a theoretical distance less than 50 nucleotides upstream of the last exon–exon junction within the mRNA, we conclude that the mutated transcript could escape the NMD process [4]. However, the patient’s fibroblasts were not available to study the outcome of this variant at the mRNA and protein levels. We can hypothesize that a proportion of mutated transcripts acting as null alleles can be translated into a shortened protein that does not function properly. 

Notably, a segregation study in the proband’s parents showed a very small peak in the Sanger chromatogram of the clinically asymptomatic father, and this suggested the presence of mosaicism for the daughter’s variant in the latter. Then, the mosaicism was confirmed by the NGS analysis and was estimated to be 4.8% in the peripheral blood by the ddPCR analysis. The mosaicism was also detected in other tissues of the patient’s father, including saliva, hair bulbs and nails. The findings corroborated the hypothesis that the mosaic variant arose as a post-zygotic mitotic event in the father and could be an example of gonosomal mosaicism. Mosaicism is a well-established biological phenomenon. Depending on the developmental stage in which a variant arises, mosaicism can be divided into one of three categories: (i) germline mosaicism (also known as gonadal mosaicism), (ii) somatic mosaicism or (iii) gonosomal mosaicism (a combination of germline and somatic mosaicism) [13]. The mosaic variant may be limited to a specific tissue and level of mosaicism vary among tissues. Major classes of mosaic disorders are known to be inherited in an autosomal-dominant manner. However, the rate of mosaicism seems to vary among different disorders and depends on the prevalence of de novo cases: the higher the prevalence of de novo cases, the higher the frequency of mosaicism should be. In the field of hereditary connective tissue disorders, the frequency of mosaic is approximately 16% in osteogenesis imperfecta [8], while only a few cases were reported on Marfan syndrome [14].

Failure to identify low levels of mosaic variants may cause a misinterpretation of genetic testing data. In particular, an inherited case may be misinterpreted as sporadic due to low-level mosaicism in a carrier parent. Of consequence, this misinterpretation may affect the recurrence risk assessment in comparison to de novo events. Thus, the identification of mosaic variants in the proband or her/his parents is relevant in a recurrence risk assessment and, hence, crucial for accurate genetic counseling and disease management. Therefore, when the apparently de novo variant is identified in a family study, the use of high-sensitivity methods, such as high-depth NGS and ddPCR, are essential to detecting low-level mosaic variants and providing an accurate assessment of the recurrence risk [6,9,14]. Genome sequencing studies allowed to evaluate the extent of mosaicism for de novo variants. A trio-based genome sequencing study on intellectual disability showed that 6.5% of de novo pathogenic variants occurred post-zygotically in the affected offspring, while an additional 0.1% of apparently de novo pathogenic variants resulted indeed from low-level mosaicism in one of the unaffected parents, thus suggesting that our genomes might be much more dynamic than previously considered [15]. More attention on mosaicism thanks to the resources of the novel technologies also allows to increase the rate of “positive results” in the probands, with potentially relevant consequences in their relatives. For example, a targeted NGS analysis identified a first case of the mosaic *NOTCH3* pathogenic variant with an allelic fraction in the leucocyte DNA of 13% in the affected proband, while this variant was subsequently demonstrated by his still unaffected two daughters, who were therefore identified as pre-symptomatic carriers [16]. 

To our knowledge, in the field of EDS, mosaicism was only occasionally reported in vascular EDS [17] and in a single family with cEDS [9]. Vascular EDS, a rare inherited autosomal-dominant disorder caused by *COL3A1* pathogenic variants, is characterized by a high frequency of de novo variants. Recently, Legrand et al. (2019) [17] screened *COL3A1* somatic mosaicism by deep-targeted NGS in 54 families and identified only one *COL3A1* case of blood mosaicism in a mother with several diagnostic minor criteria. The study estimated the frequency of *COL3A1* somatic mosaicism at 2 to 3%, suggesting this mechanism is rare but real in vEDS. More recent, Yokoi et al. (2021) [18] identified a mosaicism of *COL3A1* in a clinically asymptomatic mother of siblings with vascular EDS estimated at 6% by NGS. Very recently, Chesneau et al. (2021) [8] reported the first case of an individual with somatogonadal mosaicism in *COL5A1* identified by NGS. Although the mosaicism was estimated with a frequency of 10–15% in the blood, a clinical examination did not reveal any signs of cEDS in the father. Finally, somatic mosaicism for a *COL1A1* heterozygous pathogenic variant absent in the peripheral blood was identified in an adult with osteogenesis imperfecta/Ehlers-Danlos syndrome overlap [19]. Taken together, these data demonstrate that the introduction of NGS technology in the laboratory diagnostics of EDS and related disorders are documenting an unexpected occurrence of mosaicism in real cases. The clinical implications of mosaicism are variable and strongly influenced by the experiences of the clinical provider and diagnostic laboratory. In the absence of validated procedures, confirmation of the mosaic state with a second molecular approach and, if requested, in other tissues is mandatory for appropriate counseling. 

In summary, this study reported the second case of gonosomal mosaicism for a *COL5A1* deleterious variant in an unaffected father identified by a segregation study after identification of the same variant in the affected child. These results suggest that, in apparently de novo cases of cEDS with a documented deleterious variant, the chance of parental gonosomal mosaicism may not be low, and procedures aimed at excluding this mechanism might be considered, at least in specific cases. Further observations will allow to more accurately estimate the relevance of this phenomenon in cEDS.

## Data Availability

Not applicable.
